# Developing an AI-Assisted Educational Chatbot for Radiotherapy Using the IBM Watson Assistant Platform

**DOI:** 10.3390/healthcare11172417

**Published:** 2023-08-29

**Authors:** James C. L. Chow, Valerie Wong, Leslie Sanders, Kay Li

**Affiliations:** 1 Radiation Medicine Program, Princess Margaret Cancer Centre, University Health Network, Toronto, ON M5G 1X6, Canada; 2 Department of Radiation Oncology, University of Toronto, Toronto, ON M5T 1P5, Canada; 3 Department of Physics, Toronto Metropolitan University, Toronto, ON M5B 2K3, Canada; valerie.wong@torontomu.ca; 4 Department of Humanities, York University, Toronto, ON M3J 1P3, Canada; leslie@yorku.ca; 5 Department of English, University of Toronto, Toronto, ON M5R 2M8, Canada; kay.li@utoronto.ca

**Keywords:** medical chatbot, artificial intelligence, radiotherapy, patient education, healthcare, ChatGPT

## Abstract

*Objectives*: This study aims to make radiotherapy knowledge regarding healthcare accessible to the general public by developing an AI-powered chatbot. The interactive nature of the chatbot is expected to facilitate better understanding of information on radiotherapy through communication with users. *Methods*: Using the IBM Watson Assistant platform on IBM Cloud, the chatbot was constructed following a pre-designed flowchart that outlines the conversation flow. This approach ensured the development of the chatbot with a clear mindset and allowed for effective tracking of the conversation. The chatbot is equipped to furnish users with information and quizzes on radiotherapy to assess their understanding of the subject. *Results*: By adopting a question-and-answer approach, the chatbot can engage in human-like communication with users seeking information about radiotherapy. As some users may feel anxious and struggle to articulate their queries, the chatbot is designed to be user-friendly and reassuring, providing a list of questions for the user to choose from. Feedback on the chatbot’s content was mostly positive, despite a few limitations. The chatbot performed well and successfully conveyed knowledge as intended. *Conclusions*: There is a need to enhance the chatbot’s conversation approach to improve user interaction. Including translation capabilities to cater to individuals with different first languages would also be advantageous. Lastly, the newly launched ChatGPT could potentially be developed into a medical chatbot to facilitate knowledge transfer.

## 1. Introduction

This study focuses on developing an artificial intelligence (AI)-assisted educational chatbot for interactive learning in regard to radiotherapy. The chatbot’s primary goal is to facilitate the knowledge transfer of radiotherapy to the general public. Despite being a common treatment method for cancer since the introduction of X-ray, the general public has limited knowledge about radiotherapy beyond its association with cancer treatment. Many individuals harbor concerns regarding the safety of radiotherapy and its potential risks, despite being aware that professionals monitor the process. The study aims to provide an interactive method by developing a chatbot capable of teaching the public about the safety and efficacy of radiotherapy. Furthermore, the interactive learning technique involving conversing with a chatbot is believed to be an effective method of knowledge acquisition that does not require rote memorization [1,2,3,4]. Additionally, efforts are being made to make the chatbot sound more human in order to foster a sense of peer-like conversation with users [5,6,7,8]. The study also seeks to address the challenge of conveying radiotherapy information to individuals with varying levels of comprehension. The chatbot is designed to be user-friendly and provide straightforward explanations in response to user inquiries about radiotherapy. The chatbot’s tone is intended to be casual to create a friendly, human-like environment that reduces user stress associated with radiotherapy.

The advancement of technology has led to the development of various treatments in healthcare, beyond traditional medications and surgical procedures. For instance, the discovery of X-rays in 1895 facilitated the development of radiotherapy [9,10]. Today, radiotherapy is categorized into two main types: internal and external. However, the general public has limited knowledge about radiotherapy, its applications, and its delivery mechanisms. Therefore, this study simplifies the information regarding radiotherapy to enable easier comprehension by the general public. Internal radiotherapy, also known as brachytherapy, involves the implantation of a radioactive source directly into the target tumor or its adjacent area [11,12]. It is commonly used to treat cancers such as prostate and breast cancers. The radioactive source is enclosed in a small capsule shaped like a seed, which is then implanted using an applicator. However, this therapy can cause side effects in surrounding organs, as the radiation emitted from the source changes as it passes through different tissues. Additionally, there are various types of implantation methods, including intra-cavity and interstitial, making it vital to calculate the precise dosage required to eliminate the target tumor, as well as to determine the location for implanting the source [13].

Unlike brachytherapy, external beam radiation therapy does not require any implantation and involves direct irradiation to the target area using either photon radiation or particle radiation. Different instruments, such as a medical linear accelerator and a gamma knife, are used to perform this treatment, which has evolved into a number of techniques used to treat corresponding cancer types [14,15,16]. However, precise calculations of the radiation dose and simulations of treatment are required before any actual treatment is performed on the patient. This preparation process, known as treatment planning, is crucial to ensure the safety and accuracy of the treatment [17,18]. While it is important for professionals to understand the treatment planning process, the general public is more interested in understanding what radiotherapy is and how it is performed. Therefore, the information provided in this study focuses on transferring surface knowledge of radiotherapy, rather than going into the complicated details of the treatment planning process.

In recent years, traditional chatbots have proven to be limited in their ability to effectively manage conversations within the context of social interactions. Recognizing this drawback, Augello et al. [19] suggested a groundbreaking solution—a social chatbot model capable of integrating both individual and social processes. By considering the intricacies of human communication, this innovative chatbot model opens up new avenues for more natural and meaningful interactions between users and the AI system. The social chatbot model of Augello et al. found practical application in the development of an educational game designed to foster communicative skills learning. Drawing inspiration from literary works, this model created a verbal virtual environment that enriched the learning experience while optimizing resource utilization. This pioneering approach marked a significant step towards enhancing the potential of chatbots in educational settings.

The application of chatbot technology extends beyond the realm of education into the field of healthcare. Bibault et al. [20] delved into the use of chatbots in cancer treatment, uncovering their capacity to facilitate bidirectional information exchange with patients. This discovery opened up promising possibilities for leveraging chatbots in various aspects of cancer treatment, including patient support, monitoring, and follow-up procedures. With the ability to be deployed across multiple modalities, such as text messaging, mobile applications, and chat rooms, these AI-driven chatbots have the potential to revolutionize patient care. Furthermore, in regard to healthcare, Chung et al. [21] introduced a chatbot-based service with a knowledge base grounded in cloud computing. The service aimed to provide timely assistance to patients with chronic diseases, responding promptly to emergencies or changes in health conditions that may occur in their daily lives. Such applications of chatbot technology could significantly improve patient outcomes and bridge gaps in healthcare accessibility.

For specific medical conditions, Lokman et al. [22] devised a chatbot functioning as a virtual diabetes physician. This specialized chatbot offered diabetic patients valuable advice and support, without requiring them to visit a hospital. By enabling remote guidance and management, this AI-driven chatbot exemplified the potential of technology to enhance healthcare accessibility and efficiency. Moreover, the evolution of chatbots extends beyond healthcare into various other domains, with Hiremath et al. [23] proposing a chatbot designed not only to answer user queries comprehensively, but also to draw from both local and web databases for educational purposes. Leveraging cutting-edge technologies like machine learning, natural language processing, pattern matching, and data processing algorithms, their chatbot aimed to optimize performance and cater to diverse user needs.

In the domain of knowledge-based interactions, Setiaji et al. [24] presented a human-to-machine conversation model utilizing a relational database. The core of their chatbot accessed this database, storing knowledge, while the interface facilitated interaction by employing function and procedure sets for pattern-matching requirements. This framework showcased the potential of chatbots to harness vast repositories of knowledge and provide personalized, context-sensitive responses.

In the realm of education, the integration of chatbot technology has displayed significant promise. Heller et al. [25] developed Freudbot, a chatbot designed to explore the impact of chatbot technology on student-content interaction in distance education. Their study revealed encouraging results, suggesting that famous person applications for chatbot technology could positively influence teaching and learning experiences in distance and online education. Clarizia et al. [26] advanced the application of chatbots in education by presenting a prototype that employed advanced artificial intelligence techniques, such as natural language processing and domain ontologies. Through an experimental campaign, they demonstrated the potential of AI-powered chatbots to facilitate effective knowledge transfer in educational settings. Building on this foundation, Georgescu et al. [27] highlighted the immense potential of chatbots as cost-effective and user-friendly educational tools. Their insights underscored the versatility of chatbot technology, making it suitable for a wide range of educational scenarios, including schools, universities, and specialized training programs.

This study aims to develop an AI-powered chatbot that can educate the public, which exhibits varying levels of knowledge, about radiotherapy. To accomplish this, the chatbot will be built on IBM Watson Cloud, a platform that facilitates the creation of chatbots [28]. Based on the question-and-answer approach, the chatbot will respond to users’ questions and provide them with simple explanations in a conversational tone, thereby alleviating any anxiety they may have about radiotherapy. The chatbot’s purpose is to enhance public awareness about radiotherapy, with a focus on those who may undergo this treatment in the future, or who are generally curious about it.

## 2. Materials and Methods

### 2.1. Dialogue Flowchart

**Figure 1:**
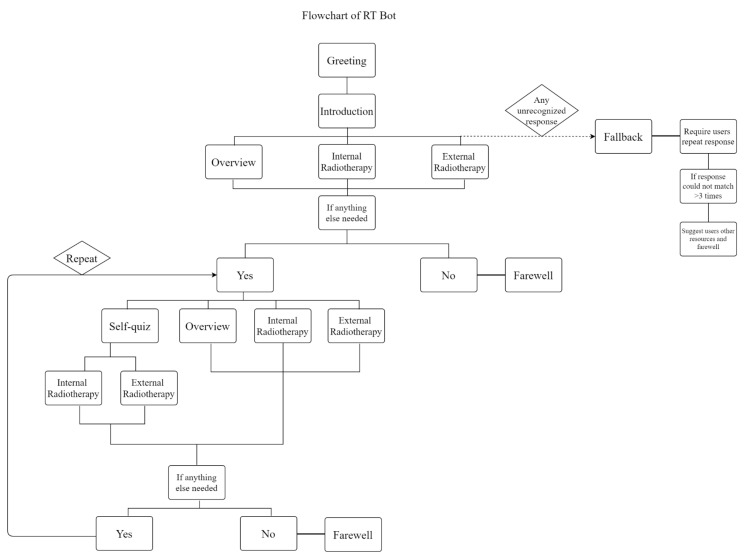
Flowchart of chatbot dialogue.

The initial step in creating the chatbot is to develop a dialogue flowchart to determine the necessary topics for discussion. This ensures that the chatbot can effectively introduce and explain radiotherapy to the general public, while maintaining an organized and user-friendly conversation. The chatbot’s main topics are reviewing radiotherapy, providing information on internal and external radiotherapies, and offering self-quizzes, as shown in Figure 1. The chatbot’s dialogue flow begins by greeting users and presenting its capabilities. Users are then presented with a list of topic options to choose from. During the conversation, the chatbot periodically checks to determine whether the user requires further information or wants to continue with the current topic. This allows users to easily end the conversation at any time, without abruptly closing the chatbot. If the user wishes to explore additional topics, the chatbot presents the list of topics again, along with the option to take a self-quiz. This process repeats until the user no longer desires further information. It is important to emphasize that the flowchart serves primarily as a starting point to set up the chatbot. Further concerns, such as the notable risk associated with infinite loops, will be dealt with during the subsequent debugging process.

### 2.2. Creating an AI-Assisted Chatbot

The IBM Watson Assistant platform was chosen to create the chatbot because the basic feature of the platform is free and allows users to create and deploy their own chatbots on various platforms [6]. The IBM Watson Assistant also offers a new feature that allows for the creation of an actions-based assistant, instead of a dialogue-based assistant built with “intents”, and this feature is utilized in this study. In addition, the chatbot built on the IBM Watson Assistant platform is AI-assisted and utilizes machine learning capabilities such as NLP [29,30,31]. By using NLP, the chatbot is capable of learning and understanding natural language questions posed by users.

### 2.3. IBM Watson Assistant Application Programming Interface (API)

**Figure 2:**
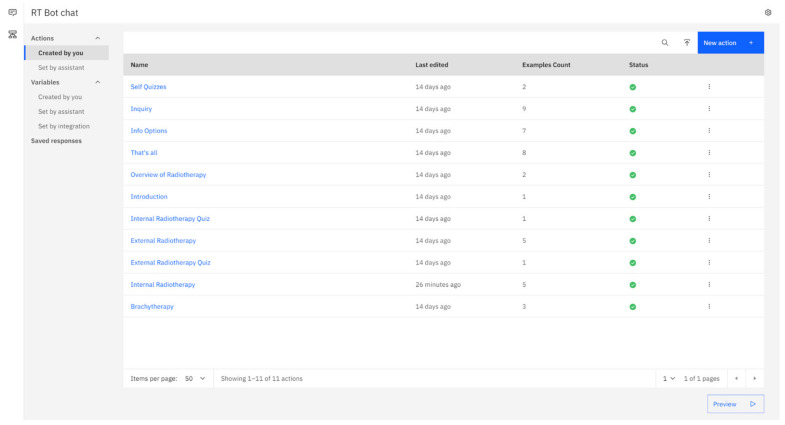
Page showing the list of actions created using the IBM Watson API.

The chatbot developed for the study is called RT Bot and is designed as an actions-based assistant. Essentially, RT Bot is equipped with a set of “actions” that enable it to respond to users’ queries. These actions can be thought of as conversation starters for RT Bot. A list of the created actions is presented in Figure 2. To add a new action, users need to select the blue “new action+” button located on the top right corner under the “created by you” tab on the left side of the page.

**Figure 3:**
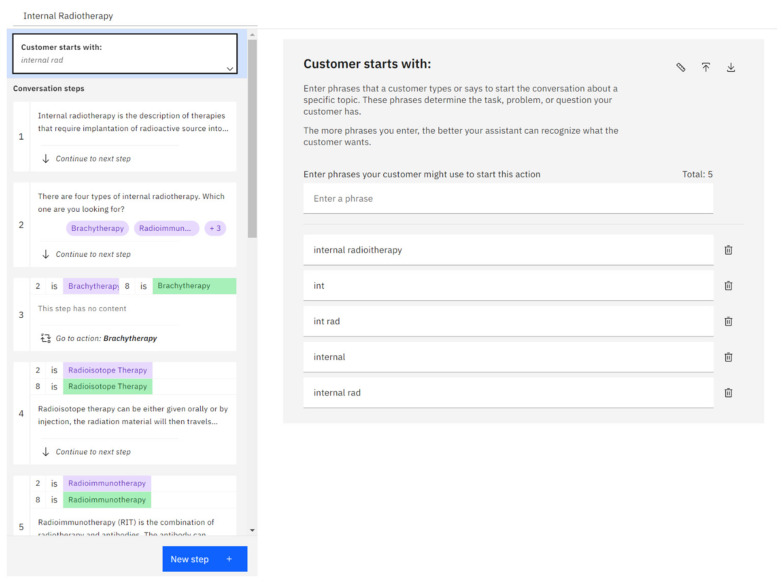
Application programing interfaces showing the training of the chatbot with possible phases.

After selecting the new action, creators will be presented with two options: “start from scratch” and “quick start with template”. They can choose to build the chatbot topic from scratch or modify an existing template provided by IBM Watson Assistant. If they select the template option, a list of 12 templates for different topics, some specific to certain industries, will be displayed. However, for RT Bot, all the topics were created from scratch. Upon selecting “start from scratch”, creators will be directed to a new page where they are prompted to define the questions or answers that the chatbot should expect from users to initiate the new topic. Moreover, the chatbot can be trained to understand the user’s responses to trigger the corresponding topics, as shown in Figure 3. The section labeled as “customer starts with” can be located at the top right side of the created topic. Creators can input phrases that users are likely to use in order to initiate the desired topic, predicting or assuming how users will request information from the chatbot. By doing this, the chatbot can comprehend and respond to various responses from different users regarding the same topic.

### 2.4. Conversation and Response

When creators define the trigger for a topic based on user responses, the dialogue flow for the chatbot (Figure 1) will be used once the “action” or topic is triggered by user responses. The chatbot will follow the conversation steps and reply to users using the script written in the API. When a user responds, the conversation will be halted until his or her response is defined. This can be accomplished by selecting the response type from the API, which includes different response types such as numbers, free text, confirmation, and lists of options. The response type restricts the user’s ability to respond to the chatbot’s questions. For example, if the response type is set to “number”, users can only respond with a numerical value. If the response type is set to “list of options”, users can only choose an option from the list. However, users can type in other responses to steer the conversation in a different direction if the options provided do not meet their needs. Apart from response types, the chatbot can transition to another topic, if the next action is selected. Additionally, it is possible to establish conditions to move to a specific conversation step within the topic. In order to enable the chatbot to jump between conversation steps within a topic, conditions can be established for each step. To accomplish this, the step is designated to be executed with conditions, and the condition can be based on the user’s response in previous steps. For instance, if the response type in the preceding step is set as “confirmation” from the user, the condition can be established such that if the user’s response is “yes”, the step will be activated. In addition to setting up conditions for individual conversation steps, it is also possible to do so for the entire topic. To create a more amiable chatbot persona, it is possible to personalize the chatbot’s interactions by addressing users by name. This can be achieved by prompting users to provide their name, which is then stored as the value of a previously established variable. The IBM Watson Assistant platform has pre-set two actions to handle situations in which the chatbot is unable to recognize the users’ responses: “no action matches” and ‘”fallback”. If the user’s response does not match any of the predetermined topics, the chatbot will initiate the “no action matches” action. This involves requesting the user to repeat their response. If the chatbot is unable to understand the user’s response after three attempts, it will proceed to the “fallback” action. The chatbot will tactfully suggest other resources to the user, with apologies.

### 2.5. Previewing and Deploying the Chatbot

In addition to testing the chatbot before its deployment, it is equally crucial to assess its performance post-deployment, through a preliminary test phase. To achieve this, the RT Bot was deployed to a website and shared with a group of 20 individuals for testing purposes. These testers lacked a scientific background, prompting an evaluation of both the chatbot’s functionality and the comprehensibility of its content. Furthermore, these testers were requested to provide feedback aimed at gauging their perceptions of the chatbot’s efficacy and its informational value. The collected comments from the feedbacks were analyzed to gain insights for enhancements of the chatbot.

In creating the chatbot in the IBM Watson Assistant platform, the graphical interface of the chatbot can be previewed on the API. This preview accurately displays how the created chatbot will appear and enables the creator to test it by responding as a user prior to deployment. This process helped the creator to verify and evaluate the performance of the chatbot under different scenarios. Once the chatbot is built and tested, it is deployed on a pre-created webpage. Apart from Internet webpage, the chatbot can be embedded on other platforms, such as Facebook Messenger, WhatsApp, and SMS, which can be found in the API.

## 3. Results

**Figure 4:**
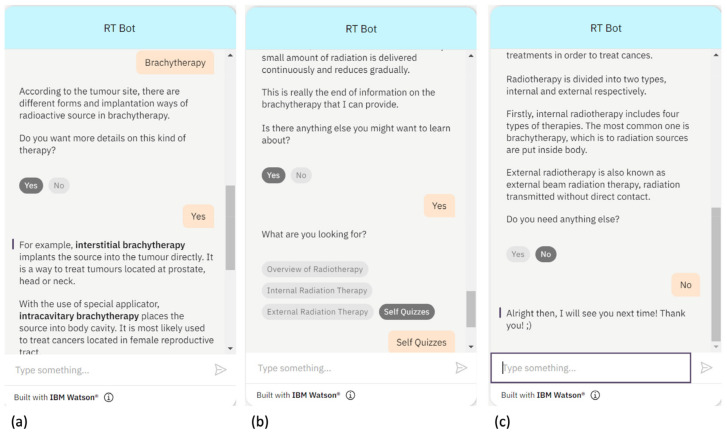
Demonstrations of the RT Bot (**a**) continuing to provide similar therapy information, (**b**) responding to the user who requires additional information after completing a section, and (**c**) saying farewell to the user.

The functionality of the RT Bot can be shown as follows: upon initiating a conversation, the chatbot greets the user, as per the dialogue flow in Figure 1, and asks for the user’s name. The chatbot then uses the provided name to address the user. In Figure 4a, the chatbot demonstrates its ability to provide information on brachytherapy. Upon receiving the user’s request, the chatbot gives a brief description of the treatment and asks the user to confirm whether or not they want to learn more. If the user responds with “yes”, the chatbot proceeds to provide more detailed information on brachytherapy. Next, after providing the previously selected information, RT Bot asks if the user wants to explore other information. If the user answers “yes”, the chatbot presents a list of information options to choose from (Figure 4b). On the other hand, if the user answered “no”, the chatbot will then say farewell to the user, as shown in Figure 4c.

**Figure 5:**
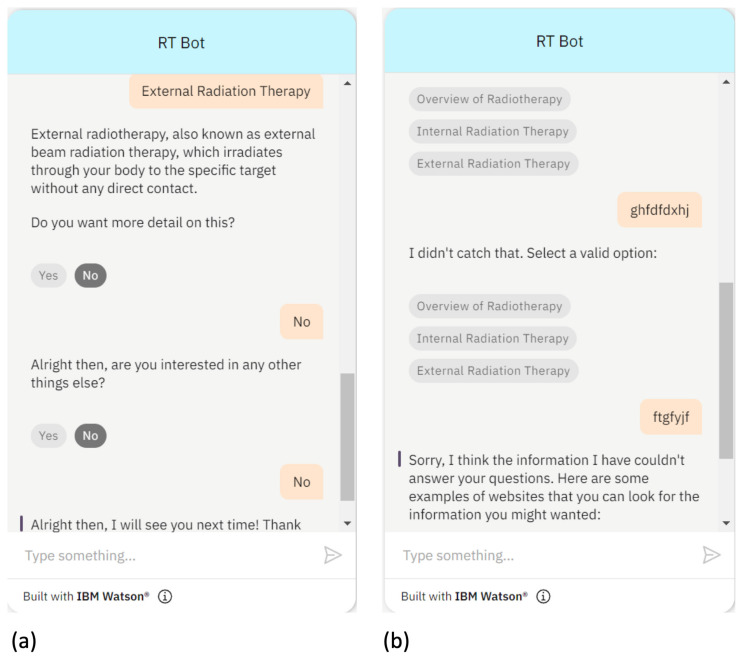
Demonstration of (**a**) ceasing receiving information on the same type of therapy and (**b**) triggering the fallback action.

Alternatively, if the user chooses to stop receiving information regarding the same therapy by answering “no”, the RT Bot will inquire whether the users wants to check other information, as shown in Figure 5a, when “external radiotherapy” is chosen. If the user also chooses “no” for checking other information, the RT Bot will say farewell to the user. In addition, if the user provides a response that does not match any of the available topics, RT Bot will ask him or her to choose from the options. If the user persists in providing responses that are out of scope or meaningless, such as random letters, RT Bot suggests that the user consult other websites for references and bids them farewell. Figure 5b demonstrates the fallback action being triggered after three unmatched responses, with RT Bot tactfully apologizing and suggesting other resources to the user.

Table 1 illustrates the users’ varying levels of content satisfaction with the RT Bot during the testing of the chatbot. In this assessment, a scale of “1” to “5” is used, with “1” indicating the lowest satisfaction, and “5” denoting the highest. The data reveal that a significant 70% of respondents perceive the content’s helpfulness, as well as its understandability and reading duration, to be above average.

## 4. Discussion

Table 1 depicts user viewpoints regarding the length of information provided by RT Bot. This evaluation aims to determine whether the information is excessively lengthy, adequately long, or too brief. The chart reflects that a significant 95% of users found the information to be sufficiently long, while 5% considered it to be overly lengthy. Remarkably, no users perceive the information as being too brief. The RT Bot has also been evaluated by different users at various workshops and conferences. Based on their feedbacks, most users had a positive opinion of the content provided by RT Bot. While these results are subjective, they align with the aim of creating the chatbot with the goal of providing knowledge in a more casual way, without overwhelming the users with academic jargon. Some users suggested improvements to RT Bot, such as showing correct quiz answers and adding a “go back” button to return to previous sessions. The former suggestion allows users to check their answers and identify areas where they need more help. This can be implemented by adding new conversation steps after the quiz results and confirming whether the user wants to review their answers. The latter suggestion would enable users to return to previous sessions without going through the entire session again. However, implementing this feature in every conversation step may make RT Bot seem annoying or less human-like, so it is being evaluated with caution.

Although most users who tested RT Bot are satisfied with its functionality, there is always room for improvement. Several ideas could potentially make the chatbot even better. One such idea is to add a translation feature for different languages, given that Canada is a multilingual country. This would allow the chatbot to transfer knowledge about radiotherapy to a wider audience. However, while the chatbot can learn and understand natural language from users, it currently only provides scripted answers, unlike ChatGPT, which can provide answers from a database [32]. Another potential improvement would be to take a more conversational approach to transferring knowledge, rather than just providing users with options to choose from. This could make interacting with the chatbot feel more like participating in real conversation and help users feel more comfortable interacting with it. In turn, this could enhance their learning experience and help them better absorb information. It is believed that ChatGPT could be used to develop a healthcare chatbot that sounds more human [33]. This could be beneficial for patients, who could talk with the chatbot before receiving treatment and learn more about radiotherapy, potentially reducing stress and anxiety. However, there are some obstacles to overcome before ChatGPT, as a disruptive technology, will be able to become a future qualified medical chatbot [33,34].

There are several limitations that hinder the creation of an ideal healthcare educational chatbot, as observed in this study. The chatbot can only provide scripted responses, even with the aid of NLP to interpret varied phrasing from users. This is due to the fact that the chatbot is created through an application that simplifies the process of chatbot creation, as opposed to the traditional method of programming with codes [28]. The templates available in the application are more suitable for customer service in different industries, rather than for informative purposes. Consequently, the RT Bot’s responses are limited to the pre-scripted patterns, making it sound less human-like. Furthermore, the chatbot created with Watson Assistant lacks the ability to perform calculations, preventing it from displaying the user’s score on the quizzes [35]. Instead, it can only encourage users if they did not receive full marks on the quiz. This limitation was also present in a chatbot created with the same developer, as noted in [6]. However, it is hoped that the developer will add this feature in future updates, allowing the chatbot to function slightly better.

It is evident that the AI-assisted chatbot is becoming increasingly popular in various industries, such as healthcare and online customer services. This chatbot is programmed with a set of flowcharts that predicts conversation between the chatbot and users, allowing it to interact with users and provide related information to answer their questions [36]. In this study, a chatbot was created to transfer knowledge regarding radiotherapy to the general public. IBM Watson Assistant was used to build the chatbot, which was designed to provide information to everyone who uses it and reduce the need for human resources to answer similar questions from different people. While some studies suggest that chatbots may not be suitable for healthcare due to their inability to recognize users’ emotions [37], other research indicates that they can be useful in transferring medical knowledge to the general public and even for improving mental health [38]. The chatbot in this study uses machine learning (ML) and natural language processing (NLP) to learn and understand the natural language of users [39]. It matches user inputs into its database using pattern similarity and linguistic analyses. OpenAI recently launched a generative language model tool called ChatGPT, which uses a new type of AI algorithm known as LLM [40]. To avoid any misunderstanding, it is important to recognize that LLMs are not standalone algorithms. Instead, they fall under a specific category of models derived from the overarching framework of the transformer architecture. This architecture, notable for its incorporation of the self-attention mechanism, serves as the foundational framework for LLMs, placing them within a broader landscape of advancements in natural language processing. While medical professionals have raised concerns about its potential to provide inaccurate medical resources, they also see its potential as a future medical chatbot if provided with appropriate training datasets [39].

At present, using LLMs in medical chatbots presents certain limitations that require careful consideration. One significant constraint revolves around the potential lack of domain-specific expertise inherent in LLMs. Medical inquiries demand a level of accuracy and precision that may not be guaranteed by general-purpose LLMs, possibly resulting in inaccurate advice or information [33]. Moreover, medical conversations often entail nuanced contexts that LLMs might struggle to fully comprehend, leading to potential misinterpretation of symptoms or queries. Ethical concerns also arise, as medical information is sensitive and demands responsible handling. LLMs could inadvertently generate biased, inappropriate, or harmful responses, highlighting the need for vigilant ethical oversight. In addition, the interactive nature of medical discussions might challenge LLMs’ ability to effectively engage in meaningful back-and-forth exchanges. In a field where data privacy, validation, and regulation are paramount, relying solely on LLMs might raise concerns about confidentiality, reliability, and adherence to medical standards. Given these limitations, a balanced approach that combines LLMs with specialized medical knowledge, human supervision, and stringent ethical practices is essential to ensure the creation of trustworthy and dependable medical chatbot systems [33].

Interactive learning aims to facilitate the stress-free and easy acquisition of new knowledge. Research suggests that communication with peers and chatbots can aid in knowledge transfer, such as in the case of learning about radiotherapy through conversations with a chatbot [41]. Rather than relying on rote memorization, chatbots interaction allows for the enhancement of the understanding of materials, according to studies on interactive learning with chatbots. Furthermore, the use of chatbots can save time by providing immediate responses to user inquiries, such as for doctor consultations or course instructor meetings. Chatbots are also a voluntary tool for users, not a mandatory learning method, and their conversational interface facilitates absorption of new knowledge in a user-friendly way. Studies have shown that applying chatbots in knowledge transfer can significantly improve the user’s understanding of information [42].

## 5. Conclusions

It is concluded that the RT Bot adequately created performs for disseminating healthcare knowledge to the public, as demonstrated by the above results. The limitations are likely due to the current process used to create the chatbot, which impacted its functionality, to some extent. Several improvements have been proposed to enhance the chatbot’s performance and provide a better conversational experience, such as incorporating additional features like language translation or text-to-speech conversion, exploring alternative conversational approaches for knowledge transfer, and integrating ChatGPT. A healthcare chatbot has the potential to assist users in various ways, particularly in disseminating medical information to the general public. Upcoming endeavors will involve a structured assessment within clinical practice, focusing on both cancer patients and the general public.

## Figures and Tables

**Table 1:** Percentage of user satisfaction regarding the contents of RT Bot.

Degree of Satisfaction (1 for Lowest, 5 for Highest)	Helpfulness of Content	Understandability of Content	Reading Time Length
1	5%	0%	0%
2	0%	15%	5%
3	25%	15%	25%
4	40%	40%	30%
5	30%	30%	40%

## Data Availability

No new data were created.
